# A Miniaturized Silicon Lab-on-Chip for Integrated PCR and Hybridization Microarray for High Multiplexing Nucleic Acids Analysis

**DOI:** 10.3390/bios12080563

**Published:** 2022-07-25

**Authors:** Giorgio Ventimiglia, Massimiliano Pesaturo, Alastair Malcolm, Salvatore Petralia

**Affiliations:** 1 EM Microelectronic, 2074 Marin-Epagnier, Switzerland; giorgio.ventimiglia@emmicroelectronic.com; 2 STMicroelectronics SRL, 20041 Agrate Brianza, Italy; massimiliano.pesaturo@st.com; 3 Applied Microarrays Inc., Tempe, AZ 85284, USA; alastair@appliedmicroarrays.com; 4 Department of Drug Science and Health, University of Catania, V. S. Sofia 64, 95125 Catania, Italy

**Keywords:** lab-on-chip, DNA microarray, nucleic acid testing, silicon biosensor, hybridization

## Abstract

A silicon lab-on-chip, for the detection of nucleic acids through the integrated PCR and hybridization microarray, was developed. The silicon lab-on-chip manufactured through bio-MEMS technology is composed of two PCR microreactors (each volume 11.2 µL) and a microarray-hybridization microchamber (volume 30 µL), fluidically connected by buried bypass. It contains heaters and temperature sensors for the management and control of the temperature cycles during the PCR amplification and hybridization processes. A post-silicon process based on (i) plasmo-O2 cleaning/activation, (ii) vapor phase epoxy silanization, (iii) microarray fabrication and (iv) a protein-based passivation step was developed and fully characterized. The ssDNA microarray (4 rows × 10 columns) composed of 400 spots (spot size—70 ± 12 µm; spot-to-spot distance—130 ± 13 µm) was manufactured by piezo-dispense technology. A DNA microarray probe density in the range of 1310 to 2070 probe µm^−2^ was observed, together with a limit of detection of about 19 target µm^−2^. The performances of the silicon lab-on-chip were validated by the detection of the beta-globin gene directly from human blood. Remarkable sensitivity, multiplexing analysis and specificity were demonstrated for the detection of beta-globin and mycobacterium tuberculosis sequences.

## 1. Introduction

The miniaturization of nucleic acid analysis represents a critical step toward the development of portable systems able to offer sample-in-answer-out diagnostic analysis [1]. Rapid and reliable identification of pathogen species, such as bacterial, fungal and viral, is one of the most important functions of a diagnostic laboratory. Conventional molecular testing involves complex procedures in laboratory infrastructures and skilled personnel. However, despite the increasing number of new diagnostic platforms based on standard and innovative molecular technologies, the amplification reaction performed by PCR is still the gold-standard technique for the molecular detection of pathogenic genomes [2], integrated into miniaturized devices for the development of portable systems.

In this context, the continuous improvement of silicon MEMS (microelectromechanical systems) technology [3], the progress of fluidic sample movements [4], the development of nanomaterials [5], and the effective integration of molecular biology on hybrid miniaturized systems [6] are strongly contributing to the development of a Lab-on-Chip (LoC) able to perform nucleic acid testing for a molecular diagnostic platform [7].

The detection of nucleic acids on miniaturized LoC faces numerous challenges in point-of-care molecular diagnostics and genetic analysis [8]. The past few decades have witnessed the development of LoCs for nucleic acid detection based on PCR/microarray hybridization [9], quantitative real-time PCR (qRTPCR) [10] and a universal microarray [11]. Although revolutionary PCR-free approaches have been recently proposed, including the CRISPR-Cas technology [12], cooperative DNA triplex formation [13], the multiplexing analysis, and the rapid detection and integration of pre-processing steps are still the main limitations for the miniaturized device. Actually, very few diagnostic platforms based on miniaturized LoC are commercially available, mainly due to the lack of sensitivity, robustness and reproducibility. The following systems are noteworthy: the Revogene system developed by GenePoC, based on microfluidics lab-on-disk technology that integrates the DNA extraction/purification processes with the real-time-PCR technology [14]; the diagnostic Cobas^®^ Liat PCR system, developed by ROCHE for the detection and identification of pathogen bacteria such as Influenza A/B, and Strep A [15]; the FireflyDX system for the detection of Influenza A and B, and HPV (Aniplex) [16], Biocartis with the IDYLLA platform, based on miniaturized PCR-on-cartridge for cancer applications [17]; the Spartan cube system for infectious diseases (Genesystem) [18]; the Verigene based on microarray hybridization detection (Nanosphere) [19]; the GeneExpert Xpress platform for infectious disease (Cepheid) [20]; and the Ag Iosystem based on electrochemical RT-PCR technologies (AG Atlas Genetics) [21]. Very recently, the ABBOTT started to commercialize the ID NOW™ COVID-19 assay for the fast detection of SARS-CoV-2 virus from buccal swabs [22].

Microarray is the most versatile technology largely diffused in the analytic and diagnostic field for the detection of various analytes, including miRNA [23], DNA [24] and pesticides [25].

In this work, an innovative method based on a miniaturized silicon device for multiplexing nucleic acids analysis by PCR and microarray hybridization is presented. The silicon LoCs consist of two silicon wafers properly bonded to create two PCR microreactors fluidically connected, through buried bypass, with a hybridization detection area. The heaters and temperature sensor are created on the bottom of LoCs for the temperature cycles.

The performance of the device was investigated performing beta-globin gene detection directly by whole human blood. The multiplexing of the system was evaluated through the simultaneous amplification and detection of 14 nucleic acid sequences of a pathogen genome extracted by human biological samples. Extraction and detection were achieved via qPCR in a unique microchamber. The excellent results obtained in terms of sensitivity, multiplexing and specificity pave the way for the future development of PoC systems, realized at low cost in different areas of molecular diagnostics.

## 2. Materials and Methods

### 2.1. Silicon Lab-on-Chip Fabrication

The silicon LoC was developed by STMicroelectronics using MEMS silicon technologies and chemical post-silicon processing.

#### 2.1.1. Silicon Process

The first LoC prototypes were developed using the SIMICH (Silicon Micro Channel) platform technology by two 6-inch silicon wafers and one borosilicate glass wafer properly bonded. From 2008 the silicon LOCs were produced by MEMS technology platform SIMICH-NE (Silicon Micro Channel New Embodiment) using two 8-inch bonded Silicon wafers, the “Sensor wafer” and the “Cap wafer”.

The silicon LoC is mainly composed of two PCR silicon microreactors (volume 11.2 μL × 2) and a microarray/hybridization chamber (dimension 8 mm × 4 mm and volume 30 μL) fluidically connected throughout bypass microchannels.

The sensor wafer is a multilayered system containing the electrical active element heaters and temperature sensors to manage and control the temperature during the biological reaction and the fluidic bypass to connect the PCR microreactor to hybridization area.

The cap wafer contains the PCR microreactors, the inlets for sample loading, and the hybridization chamber.

The silicon thermal proprieties are instrumental to control the uniformity of the temperature during these biological phases. Instead, accuracy of the temperature is guaranteed by the “wafer level calibration method” carried out along the manufacturing flow in the production line.

The key of the MEMS SIMICH-NE process is the method to generate the silicon microreactors, the inlet and the bypass channels. In terms of details, using a hard mask above the sensor wafer surface through standard deposition, lithography and dry etching technologies allows for the realization of microchannels with “V” cross-section on the silicon substrate wafer, by means of a TMAH (tetramethylammonium hydroxide) etching. The TMAH silicon etching leaves the grid untouched above the channels due to the high selective etching aspect ratio; a subsequent silicon/oxide/nitride deposition closes the grid to create the channel top. A silicon/oxide deep reactive ion etching (RIE) of buried channel top layers is carried out later in the process in order to open the inlets and the outlets at the ends of the bypass buried channels.

The PCR microreactors surface were finished by a silicon-oxide layer (thickness 800 nm) to allow the further silicon post-processing steps.

The microarray/hybridization chamber consists of a bilayer system deposited on a silicon substrate: the first Al layer was placed by CVD method (1000 nm), and the second SiO_2_ layer was placed by TEOS precursor with PE-CVD technique, for a deposition time to reach a thickness of value of 850 ± 20 nm. The wafer-to-wafer bonding technology was performed using Dry Resist Film (DRF) as glue layer.

#### 2.1.2. Post-Silicon Process

The post-silicon process consists of a multi-step procedure properly designed to allow for the surface biocompatibility for PCR amplification and to prepare a microreactor surface for covalent anchoring suitable to fabricate a uniform multi-spots microarray with ssDNA probe-microarray covalently anchored into the detection area. A final protein-based blocking step was developed to passivate the microreactor surface to avoid unspecific interaction and to achieve a PCR-friendly surface. All processes were performed in a clean room (class 1000).

##### Chemical Processes

The LoCs were chemically processed as follows: (i) cleaning and activation process by plasma-O_2_ treatment for 300 s, 100 W (Sentech plasma equipment) to remove organic residues from surface and to increase the hydroxyl group density at SiO_2_ surface; (ii) vapor phase silanization to produce a reactive epoxy-silane monolayer at SiO_2_ surface (10 mL of GOPS (3-Glycidyloxypropyltrimethoxysilane)) in a vacuum-oven for 4 h, 0.1 Atm, and temperature of 125 ± 1 °C.

In order to confirm the effectiveness of the plasma-O_2_ and epoxy silanization treatments, water contact angle (CA) measurements, ellipsometry and TOF-SIMS were performed.

After O_2_-plasma process, the microarray/hybridization microchamber surface exhibits a high wettability (CA < 10°) to indicate the effective cleaning and oxidation processes. Contrastingly, after vapor phase epoxy-silanization, an increase in surface hydrophobicity was observed (CA values 56.1° ± 1.3°) to indicate the formation of the epoxy-layer coating.

The TEOS thickness ellipsometry measurements reported for the untreated detection area a TEOS thickness of about 850 ± 4 nm; after silanization, a slight increase in thickness (860 ± 4 nm) was observed to indicate the formation of the epoxy-silane on TEOS surface. The epoxy-silane coating formation on TEOS surface was confirmed by TOF-SIMS investigation. Diagnostic signals related to CxHy (3000 a.u.) and SiCxHyO (5000 a.u.) were observed at zero and after 1 sec of sputter time, respectively. These peaks drastically decrease after 5 sec of sputter time (<400 a.u.) to indicate the presence of a silicon-organic layer at TEOS surface. This was because reference LoCs were investigated after plasma-O2 treatment (without epoxy silanization process) and no significant signals related to CxHy and SiCxHyO were observed. The XP investigations confirm the effectiveness of the epoxy silane process: by the increase in the C1s signal (after Plasma-O_2_: C1s 4.0%, O1s 65.50%; after vapor phase silanization: C1s 10.2%, O1s 61.0%).

##### LoCs Microarray (LoC-MA) Fabrication

**Figure 1:**
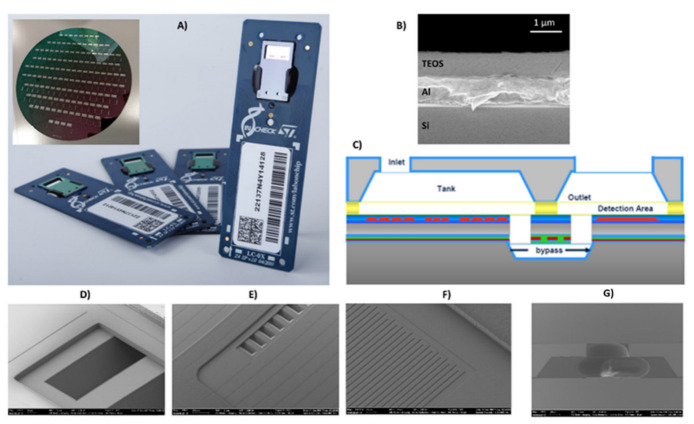
(**A**) Silicon LoC developed by STMicroelectronics (inset the silicon-cap wafer); (**B**) multilayered detection area, fluidics inlet; (**C**) schematic of the LoCs; (**D**) bypass inlet; (**E**) bypass outlet; (**F**) metal sensor; (**G**) inlet silicon cap.

The LoC-MA was fabricated by micro-piezo spotting technology using the Bio-jet equipment located at Applied Microarrays Inc. clean room facility in Tempe, AZ, USA. The Bio-jet micro-spotter was equipped with piezo-tips (orifice size of 40 µm) and a camera for the after-printing controls. The microarray layout is composed of 400 spots, positioned on 40 columns and 10 rows with a spot-pitch of 130 µm (Figure 1). The spot-position tolerance was 13 ± 6 µm. The after-printing image for HDMA-TP was acquired by Aurigin camera and the image analysis performed by MIT software v 1.0 (developed by STMicroelectronics, Agrate Brianza, Italy). The microarray geometrical parameters monitored were: spot size, spot position, array position and grid array area. The HDMA-test pattern used as microarray model is composed of three ssDNA-probe hybridization controls (ST1, ST2 and ST3), three ssDNA probes specific for human beta-globin gene (BG1, Bg2 and BG3) and empty spots to evaluate the background level (Figure S1).

**Figure 2:**
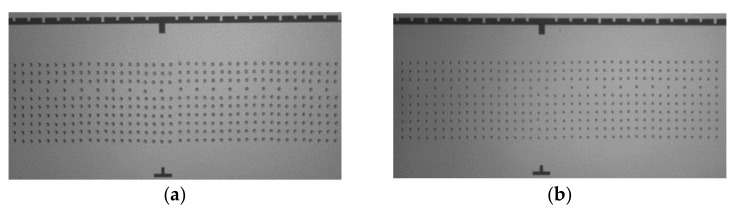
Optical image of HDMA-BG printed on LoC detection area using disodium hydrogen phosphate printing buffer (pH value 9.2) at concentration 120 mg/mL (**a**) and 50 mg/mL (**b**).

In order to investigate the HDMA spot-size, microarrays were printed using two different printing buffer compositions (disodium hydrogen phosphate 50 mg/mL and 120 mg/mL at pH value 9.2). The after-printing image analysis report spot size values of 50 ± 10 µm and 70 ± 12 µm for the printing buffer 50 and 120 mg/mL respectively (Figure 2). As expected, these data indicate that the spot size is tunable by varying the buffer composition. In order to have larger spot size and a higher anchored probes amount, the following microarray fabrication processes have been conducted using the printing buffer concentration of 120 mg/mL and pH value of 9.2.

##### Passivation Process

After vapor phase silanization process a protein-based passivation step was designed to avoid unspecific interaction between biomolecules and microreactor surface during PCR and hybridization step. The passivation process was performed dipping the LoCs on aqueous solution of bovine serum albumin (BSA) 1% p/v, SSC 2X, SDS 0.1%, for 15 h at 55 °C. The substrates were then rinsed in deionized water and dried by a nitrogen flow. The BSA-based passivation layer was indicated by the decrease in the CA values (52.0° ± 3.2°), by the variation of the ellipsometry signal (820 ± 8 nm) due to the change of the index refraction of the surface and by the XPs investigation by the increase in the diagnostic C_1s_ and N_2p_ peaks (C_1s_ 17.3%, O_1s_ 53.7%, N_1s_ 2.46%).

### 2.2. PCR Amplification Experiments

The PCR amplification performances were evaluated by complete Human beta-globine (HBG) gene, and the sequences of primers are reported in SI. DNA amplifications were performed in PCR mix aliquots of 25 μL using 4 U Hotstart Taq plus DNA polymerase, 200 μM each dNTP (dATP, dCTP, dGTP and dTTP), 1 μM primer, 1 μM human DNA, 1X PCR buffer (HotStarTaq Plus DNA Polymerase, Qiagen kit, Hilden, Germany) and sterile water on a Epperdorf Mastercycler Gradient (New York, NY, USA). PCR thermos-protocol used was denaturation step at 95 °C for 5 min and followed by 25 cycles of amplification (20 s at 95 °C, 45 s at 61 °C, 72 s at 30 °C). The PCR products and their size were measured (in the range of 15–1500 base pairs), by Agilent 2100 Bioanalyzer (Agilent, Waldbronn, Germany). Same procedure was used to test the microarray for MTB gene (Mycobacterium tuberculosis).

### 2.3. Hybridization Experiments

The hybridization experiments were carried out using Cy5-labelled target perfect-perfect match (15.0 nM) in 20 mM of sodium phosphate buffer, 1 M NaCl, 5.2 mM fo KCl, 0.1% of Tween20, 2 X of Denhardt’s solution, and 20 µg/µL of ssDNA. After hybridization, a washing step consisting of 5 min at 40 °C in SSC 2X (code SRE0068 Sigma, Kawasaki, Japan), followed by a second wash of 5 min at 40 °C in 0.2X SSC was carried out. Hybridized images have been acquired by standard Optical Reader managed by EA software, while the image analysis has been performed by H-MAT and E@sy Check software v 2.0. The optical reader, developed by STMicroelctronics, is an optical device for acquiring images of LoC. It is equipped with a camera 1392 × 1040 pixels operating in 8-bit mode and has two programmable outputs that are used for turning on an excitation system based on two identical illuminators placed symmetrically to the sides of the camera and tilted at 45°. The illuminator consists of a 5 mm white and red LED, two aspherical lenses and an excitation filter specific for Cy5. To increase the dynamic linear range, a multi-shot acquisition is used to capture the image at various exposition times, ranging from 15 to 4000 ms.

### 2.4. Integrated PCR/Hybridization Experiments

In order to evaluate the integrated PCR/hybridization process on LoC, different amounts of human DNA template were mixed with PCR reagent. Then, aliquots of 11.5 uL of PCR mix were loaded on each LoC PCR microreactor. The LoC was then sealed by specific PCR-clamps, while the chip was inserted into the TCS (Therma Cycles System) developed by STMicroelectronics and thermally cycled as follows: denaturation step at 95 °C for 5 min, which was followed by 35 cycles of amplification (20 s at 95 °C, 45 s at 61 °C, 72 s at 30 °C). Afterwards, the liquid containing the PCR product was moved by manual operation from the silicon microreactors to the microarray chamber, pipetting 11.5 uL of hybridization reagent into each micro-reactor, using a standard pipette equipped with P20-tips (Axygen, Union City, CA, USA). The LoC was then manually sealed by specific PDMS clamps, while the chip was inserted into the TCS and thermally cycled as follows: denaturation step at 95 °C for 2 min was included prior to the hybridization step at 55 °C for 30 min. After the thermal process, the LoC was removed from the TCS, and a washing step was performed in a centrifuge station, for 5 min at 40 °C in 2× SSC. The chip was dried under nitrogen and the image was acquired by the In-Check OR. The assessment of specificity was performed by including the BG-PCR-primer (1 µM) in the PCR mix, as well as the human DNA and MTB genes. The hybridization signals were recorded at 2000 ms. Similarly, the multiplexing investigation was performed including the BG-PCR-primer (1 µM) and the MTB-PCR-pimer (1 µM) in the PCR reagent mix, together with both the human and MTB genes.

In SI, the whole LoC-MA testing process flow is illustrated.

### 2.5. Detection of Beta-Globin Gene from Whole Human Blood

In order to assess the ability of LoC to detect the nucleic acids directly from real sample, the recognition of the beta-globin gene from whole human blood was performed, and the results are reported here. The first step consists of a one-pot DNA extraction process performed on a standard microtube using the prepGEM Tissue Kit (Zygem, Southampton, UK), and the second step is the integrated PCR amplification of extracted human DNA and hybridization reaction of amplicons and finally microarray fluorescence read out. The PCR, hybridization and fluorescence read out are whole integrated on LoC device.

## 3. Results and Discussion

The LoC developed by STMicroelectronics, Italy, is mainly composed of a silicon chip mounted on a PCB board (Figure 1A). The silicon chip contains two PCR microreactors and a microarray/hybridization chamber. The microarray/hybridization chamber consists of a bilayer system deposed on silicon substrate: an Al mirror layer and a SiO_2_ layer (Figure 1B). The by-layered architecture into detection area allows for the optical enhancement of the fluorescence signal for the Cy5 fluorophores (fluorescence wavelength emission about 670 nm) [26]. The two PCR microreactors were passivated by a silicon oxide layer (thickness 800 nm). The microarray/hybridization chamber is fluidically connected to the PCR microreactors by buried microchannel bypass (Figure 1C), through the inlet and the outlet bypass (Figure 1D,E). The metal sensors and heater are properly designed to manage and control the temperature during the PCR cycles and the hybridization reaction (Figure 1F). The inlet on the silicon cap permits the loading of the sample (Figure 1G).

### 3.1. LoC-MA Microarray Hybridization Testing

The LoC-MA performances in terms of nucleic acid detection were evaluated investigating the hybridization signal and the integrated PCR-hybridization results.

**Figure 3:**
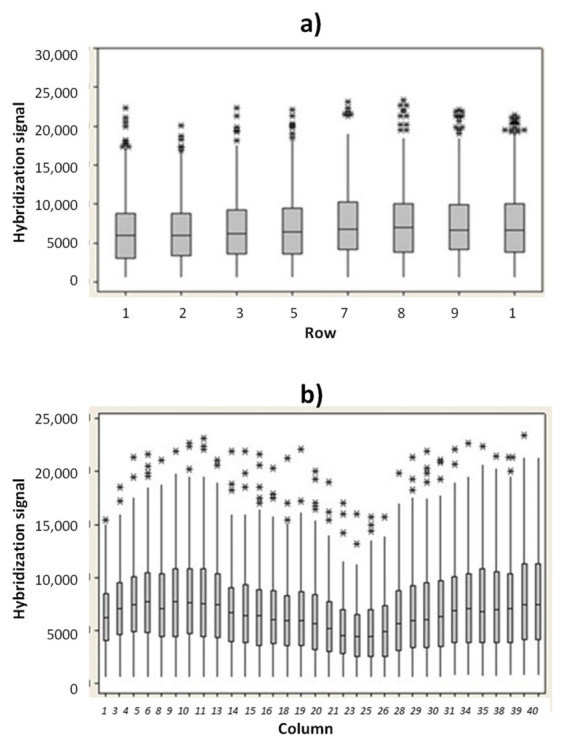
Hybridization signal of ST1 probe (acquisition time 15 ms) versus row (**a**) and versus column (**b**). (* outlier data).

In order to verify the uniformity of hybridization signal into the microarray area, the hybridization signal for the ST1 probe versus rows and columns position were evaluated. In Figure 3a are reported the ST1 hybridization fluorescence signals (median values) versus the row positions. Similarly, the hybridization signal for ST1 probe versus column positions were investigated (Figure 3b). The experimental results indicate that no drastic difference in hybridization signal occurs between the rows and the columns.

The slight decrease in hybridization signal reported in the microarray area from column 21 to 28 is related to a minor illumination. However, all the hybridization signals measured in this region (from column 21 to column 28) were higher than 5000 a.u., if captured with an exposition time value of 15 ms. A coefficient of variation (CV) value of the illumination uniformity of just 9.10% was measured (S3). A hybridization signal above 2000 a.u. (measured at 15 ms of exposition time) and a CV% value below 10% are the acceptance criteria used for the hybridization module.

**Figure 4:**
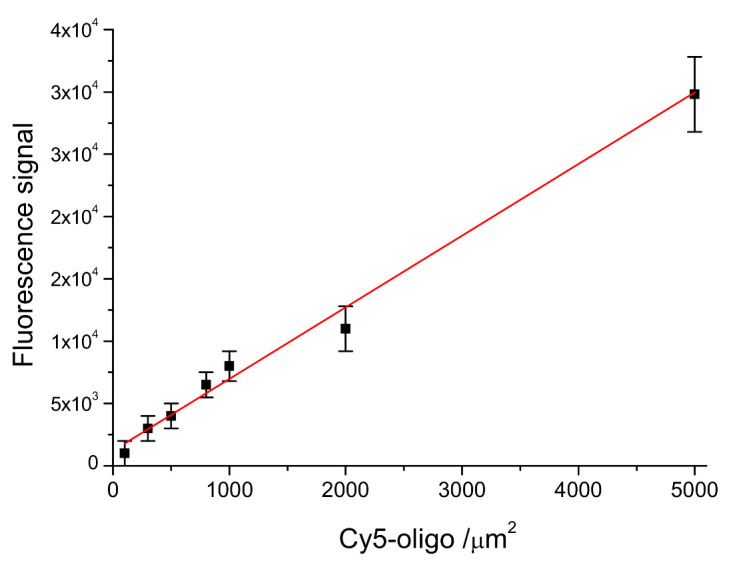
Linear correlation between the probe density (Cy5-oligo/µm^2^) versus the fluorescence signal intensity (Y = 1220 + 5.8X; R^2^ = 0.9695).

In order to evaluate the DNA-probe density on LoC-MA, the fluorescence signal of the 5′ Cy5-labeled probe anchored at the LoC-MA detection area surface has been measured. The experimental data report, for 10 LoC-MA, replicates a signal of about 11,000 ± 2.200 a.u. The related probe density value was extrapolated from the calibration line probe density versus fluorescence signal (Y = 1220 + 5.8X; R^2^ = 0.9695) as reported in Figure 4. This calibration line was prepared by spotting seven different 5′ Cy5-labeled probe amount (concentration ranging from 10 to 500 nM) on LoC-MA devices (25 replicas). The extrapolation with the regression line equation shows for the anchored probe on LoC-MA a probe density value of 1690 ± 380 probe amount µm^−2^.

Moreover, from the calibration line, the limit of detection (LoD) for the microarray capture probes has been calculated using the Y value as a current value corresponding to three-times the background signal (1312 a.u.). An LoD of about 19 target µm^−2^ was calculated.

**Figure 5:**
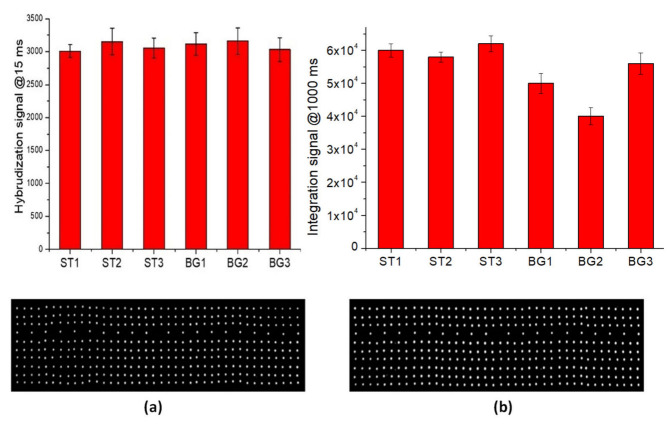
LoCs-MA testing: (**a**) hybridization experiment fluorescence signal (15 ms) for the hybridized spot (**top**) and representative fluorescence image of hybridized MA-BG (**bottom**); (**b**) Integrated experiment fluorescence signal (1000 ms) for the hybridized spot (**top**) and representative fluorescence image (**bottom**).

The hybridization tests of perfect-match oligonucleoyides were performed using the procedure reported in the Materials and Methods section. The fluorescence signal (at 15 msec of exposure time of optical reader) for all hybridized spots are illustrated in Figure 5a. The data show a good hybridization performance of LoC-MA with respect to all the probes investigated.

The integrated test, rather than the direct detection of the beta-globin gene, was performed by PCR amplification of the three specific beta-globin targets (BG1, BG2 and BG3) and the hybridization of PCR amplicon on MA. Figure 5b illustrates the fluorescence signal for BG1 (50,000 ± 3000 a.u.), BG2 (40,000 ± 3000 a.u.), and BG3 amplicons (56,000 ± 3200 a.u.), together with the fluorescence signal of the hybridization perfect match control ST1 (60,000 ± 2000 a.u.), ST2 (58,000 ± 1500 a.u.) and ST3 (62,000 ± 2400 a.u.). Due to the low hybridization signal intensity, the image acquisitions were conducted at 1000 ms of exposition time. The data indicate an excellent LoC-HDMA performances to perform the hybridization detection of BG amplicons. The low signal recorder for the BG2 probe was related to the low PCR efficiency of the BG2-PCR-primers (data confirmed by PCR experiments on microtubes). Again, the fluorescence signal for the hybridization controls (ST1, ST2 and ST3) indicate the excellent efficiency of the surface hybridization.

### 3.2. Detection of Beta-Globin Gene from Biological Sample

**Figure 6:**
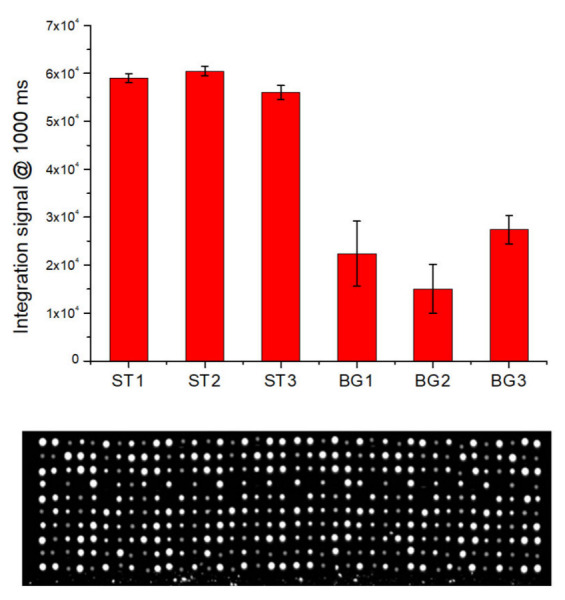
Integrated experiment for the detection of BG on whole human blood by LoC-MA: fluorescence signal (1000 ms) for the hybridized spot (**top**) and representative fluorescence image (**bottom**).

To evaluate the ability of LoC-MA to recognize nucleic acids from biological samples, the detection of the BG gene from whole human blood samples was performed. A DNA extraction procedure developed in our previous work was used [8]. In detail, 5 µL of whole human blood was mixed with 94 µL of 1× prepGEM buffer and 1 µL of prepGEM Blood Reagent. A volume of 20 µL of the resulted solution was incubated in a thermocycler. The initial step of 5 min at 75 °C was used to perform both cell lysis and proteinase digestion. This step involved proteinase inactivation for 5 min at 95 °C. After that, an aliquot of the extracted solution was added to the PCR mix, and 11.5 µL of the resulted solution was loaded for each microreactor. After the PCR cycles, the reaction mix was moved to the hybridization area and the hybridization experiment started. Figure 6 illustrates the fluorescence signal for the hybridization of the BG-gene amplicons BG1 (22,377 ± 6789 a.u.), BG2 (15,062 ± 5065 a.u.) and BG3 (27,418 ± 2928 a.u.) and the hybridization signal for the perfect match hybridization controls ST1 (59,000 ± 904 a.u.)., ST2 (60,500 ± 1010 a.u.) and ST3 (56,500 ± 1453 a.u.).

#### 3.2.1. Limit-of-Detection Assessment

The sensitivity of the integrated test assay was evaluated using serial dilutions of human DNA in the range of 0, 20, 50, 100, 250, 2500, and 5000 cps per reaction (each tested in triplicate). The fluorescence hybridization signals (BG1 probe) for the integrated test at various DNA template amounts were, respectively: 20 cps/reaction (800 ± 300 a.u.), 50 cps/reaction (950 ± 450 a.u.), 100 cps/reaction (2100 ± 500 a.u.), 250 cps/reaction (3700 ± 6500 a.u.), 2500 cps/reaction (21,500 ± 2250 a.u.) and 5000 cps/reaction (25,000 ± 2340 a.u.). The limit of detection (LoD), calculated as 3.3 × σ_backgroud_, was about 10 copies/reaction. This datum was in agreement with the LoD values (10–100 copies/reaction) obtained for previous versions of LOC [8,27].

#### 3.2.2. Multiplexing Assessment

To evaluate the multiplexing potential of the LoCs-MA as well the specificity of the assay, PCR/hybridization integrated experiments were carried out interrogating the LoCs-MA for 17 different specific targets and 3 hybridization controls. Specifically, LoCs-MA containing 3 hybridization control (ST1, ST2 and ST3), 3 specific human BG-gene probes (BG1, BG2 and BG3) and 14 specific probes for MTB were designed, prepared and tested. To assess the specificity of the assay, 10 LoCs-MA were interrogated with human DNA template and Mycobacterium tuberculosis (MTB) genome by a PCR mix containing the PCR primers specific for BG.

**Figure 7:**
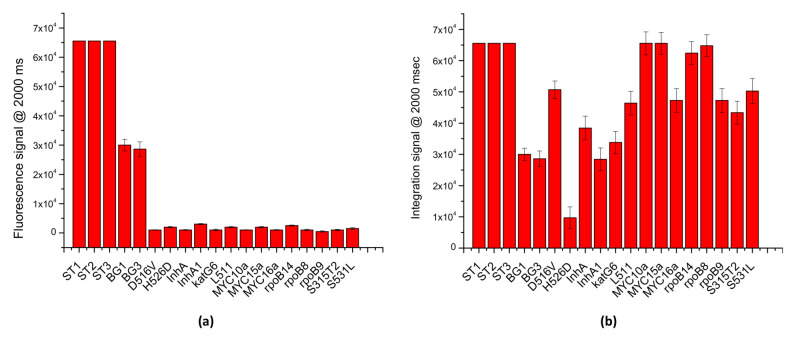
LoCs-MA integrated experiments: (**a**) specificity tests; (**b**) multiplexing analysis.

The results illustrated in Figure 7a indicate an excellent specificity of the assay, for the detection of the BG gene; indeed, just fluorescence signals for specific BG probes were recorded (BG1 30,000 ± 2000 a.u. and BG3 28,600 ± 2500), while no significant signal (<3000 a.u.) was observed for the MTB probes. Similarly, to investigate the multiplexing potential of the assay, 10 LoC-MA were interrogated with a PCR mix containing BG-PCR primers and MTB-PCR primers specific for the recognition BG and MTB genes, respectively. Due to the low signal intensity, the image acquisitions were conducted at 2000 ms of exposition time. The results illustrated in Figure 7b confirm an excellent multiplexing analysis; indeed, the graph shows intense fluorescence hybridization signals for all the 16 probes investigated (13 MTB specifics probes and 2 BG specific probes). Additionally, the fluorescence signals observed for the hybridization controls (ST1, ST2 and ST3 > 65,000 a.u.) confirm, again, the good hybridization response of the MA.

All these data confirm the excellent performance of the LOCs-MA in terms of sensitivity, specificity and mostly in multiplexing analysis for the recognition of nucleic acids. The enhanced fluorescence signal of multilayered (Si/Al/SiO_2_) detection area together with the effective integration of PCR and microarray hybridization into silicon chip allows for good sensitivity of the microarray analysis. The integrated test data indicate an excellent specificity of the LoCs-MA assay with a potential multiplexing analysis, and 15 specific sequences of two different genomes were detected in a unique analysis. At first glance, it seems that the main disadvantages of the proposed LoC are the single-sample process and the outside sample preparation. However, a critical discussion on this topic allows one to point out that the single-sample process is not a real drawback but, on the contrary, a benefit, as it limits the sample cross-contaminations. Similarly, the outside sample preparation is not fundamental for a versatile platform based on microarray, as it is meant to analyze samples with different sizes, densities, etc., such as biological specimens, water, and food for various applications.

## 4. Conclusions

In the present work, we report the development of the silicon lab-on-chip for the multiplexing detection of nucleic acids through the integrated PCR amplification and microarray hybridization reactions. The silicon lab-on-chip manufactured by STMicroelectronics, using MEMS technology, is composed of two PCR microreactors and a microarray-hybridization microchamber fluidically connected through buried bypass. A properly post-silicon process based on plasma-O_2_ cleaning/activation, vapor phase epoxy silanization, high-density microarray fabrication and a protein-based passivation step was developed, and the results are presented here. The microarray was composed of 400 spots and manufactured by piezo-array technology. The performances of the silicon lab-on-chip was investigated by the detection of the beta-globin gene directly from human blood. The sensitivity, the specificity and the potential multiplexing analysis were demonstrated by the detection of BG and MTB sequences.

## Supplementary Materials

The following supporting information can be downloaded at: https://www.mdpi.com/article/10.3390/bios12080563/s1, SI1: Microarray layouts, SI2: Reagents and methods, SI3: Fully integrated, procedure, SI4: Instrumentation, SI5: Illumination uniformity test, SI6: Calibration curve for spot-probe density, SI7: Spot size from fluorescence integrated signals for the layout BG-MTB, SI8: LoC-MA testing process flow. Figure S1: HDMA-BG layout, 400 spots (replicates: ST1 64 spots, ST2 64 spots, ST3 60 spots, BG1 64 spots, BG2 64 spots BG3 64 spots empty 20). Figure S2: HDMA-BG layout 400 spots (replicates: ST1 28 spots, ST2 22 spots, ST3 20 spots, BG1 22 spots, BG2 22 spots BG3 20 spots empty 20, 3 replicas for each MTB specific probe. Table S1: Probe and target oligonucleotides sequences. Figure S3: Illumination test for LoC-MA microarray area. Figure S4: Spot size after Integrated (PCR/hybridization) test. Figure S5: Testing process flow: DNA extraction on tube, PCR amplification and hybridization on LoC-MA, image acquisition by Optical Reader and image analysis.
