# Supplementary material for: A Miniaturized Silicon Lab-on-Chip for Integrated PCR and Hybridization Microarray for High Multiplexing Nucleic Acids Analysis
Source: Biosensors (Basel). 2022 Jul 25;12(8):563. doi: 10.3390/bios12080563 (PMC9332503; doi:10.3390/bios12080563)
Supplement: Supplementary file 1 [file biosensors-12-00563-s001.zip › biosensors-1812775-supplementary.pdf]

biosensors

Article

A Miniaturized Silicon Lab-On-Chip for Integrated PCR and Hybridization Microarray for High Multiplexing Nucleic Acids Analysis

Giorgio Ventimiglia [1], Massimiliano Pesaturo [2], Alastair Malcolm [3] and Salvatore Petralia [4,*]

[1] EM Microelectronic, Marin-Epagnier, Switzerland; giorgio.ventimiglia@emmicroelectronic.com
[2] STMicroelectronics srl, Agrate Brianza, Italy; massimiliano.pesaturo@st.com
[3] Applied Microarrays Inc., Tempe, AZ, USA; alastair@appliedmicroarrays.com
[4] Department of Drug Science and Health, University of Catania, V. S. Sofia 64, 95125 Catania, Italy
* Correspondence: salvatore.petralia@unict.it

SI1 Microarray layouts

SI2 Reagents and methods

SI3 Fully integrated procedure

SI4 Instrumentation

SI5 Illumination uniformity test.

SI6 Calibration curve for spot-probe density.

SI7 Spot size from fluorescence integrated signals for the layout BG-MTB

SI8 LoC-MA testing process flow

SI1 Microarray layouts

BG layout

	1	2	3	4	5	6	7	8	9	10	11	12	13	14	15	16	17	18	19	20	21	22	23	24	25	26	27	28	29	30	31	32	33	34	35	36	37	38	39	40
1	ST1	ST2	BG1	ST3	BG3	ST2	BG2	ST3	BG1	ST2	ST2	BG1	ST3	BG2	ST2	BG3	ST3	BG1	ST2	ST1	ST1	ST2	BG1	ST3	BG3	ST2	BG2	ST3	BG1	ST2	ST2	BG1	ST3	BG2	ST2	BG3	ST3	BG1	ST2	ST1
2	BG3	BG2	BG3	ST1	BG2	BG1	ST1	BG3	BG2	ST1	ST1	BG2	BG3	ST1	BG1	BG2	ST1	BG3	BG2	BG3	BG3	BG2	BG3	ST1	BG2	BG1	ST1	BG3	BG2	ST1	ST1	BG2	BG3	ST1	BG1	BG2	ST1	BG3	BG2	BG3
3	ST1	ST3	BG1	ST2	BG3	ST3	BG2	ST2	BG1	ST3	ST3	BG1	ST2	BG2	ST3	BG3	ST2	BG1	ST3	ST1	ST1	ST3	BG1	ST2	BG3	ST3	BG2	ST2	BG1	ST3	ST3	BG1	ST2	BG2	ST3	BG3	ST2	BG1	ST3	ST1
4	ST2	/	BG1	/	ST1	/	BG2	/	ST3	/	BG3	/	ST1	/	BG1	/	ST2	/	BG2	/	/	ST2	/	BG1	/	ST1	/	BG2	/	ST3	/	BG3	/	ST1	/	BG1	/	ST2	/	BG2
5	ST1	ST2	BG1	ST3	BG3	ST2	BG2	ST3	BG1	ST2	ST2	BG1	ST3	BG2	ST2	BG3	ST3	BG1	ST2	ST1	ST1	ST2	BG1	ST3	BG3	ST2	BG2	ST3	BG1	ST2	ST2	BG1	ST3	BG2	ST2	BG3	ST3	BG1	ST2	ST1
6	BG3	BG2	BG3	ST1	BG2	BG1	ST1	BG3	BG2	ST1	ST1	BG2	BG3	ST1	BG1	BG2	ST1	BG3	BG2	BG3	BG3	BG2	BG3	ST1	BG2	BG1	ST1	BG3	BG2	ST1	ST1	BG2	BG3	ST1	BG1	BG2	ST1	BG3	BG2	BG3
7	ST1	ST3	BG1	ST2	BG3	ST3	BG2	ST2	BG1	ST3	ST3	BG1	ST2	BG2	ST3	BG3	ST2	BG1	ST3	ST1	ST1	ST3	BG1	ST2	BG3	ST3	BG2	ST2	BG1	ST3	ST3	BG1	ST2	BG2	ST3	BG3	ST2	BG1	ST3	ST1
8	ST1	ST2	BG1	ST3	BG3	ST2	BG2	ST3	BG1	ST2	ST2	BG1	ST3	BG2	ST2	BG3	ST3	BG1	ST2	ST1	ST1	ST2	BG1	ST3	BG3	ST2	BG2	ST3	BG1	ST2	ST2	BG1	ST3	BG2	ST2	BG3	ST3	BG1	ST2	ST1
9	BG3	BG2	BG3	ST1	BG2	BG1	ST1	BG3	BG2	ST1	ST1	BG2	BG3	ST1	BG1	BG2	ST1	BG3	BG2	BG3	BG3	BG2	BG3	ST1	BG2	BG1	ST1	BG3	BG2	ST1	ST1	BG2	BG3	ST1	BG1	BG2	ST1	BG3	BG2	BG3
10	ST1	ST3	BG1	ST2	BG3	ST3	BG2	ST2	BG1	ST3	ST3	BG1	ST2	BG2	ST3	BG3	ST2	BG1	ST3	ST1	ST1	ST3	BG1	ST2	BG3	ST3	BG2	ST2	BG1	ST3	ST3	BG1	ST2	BG2	ST3	BG3	ST2	BG1	ST3	ST1

Figure S1 HDMA-BG layout, 400 spots (replicates: ST1 64 spots, ST2 64 spots, ST3 60 spots, BG1 64 spots, BG2 64 spots BG3 64 spots empty 20)

BG-MTB layout

	1	2	3	4	5	6	7	8	9	10	11	12	13	14	15	16	17	18	19	20
1	ST1	ST2	H526_w14	AT809	Empty	rpoB8	Empty	Empty	rpoB14	BG2	BG3	Empty	BG1	Empty	Empty	Empty	BG3	Empty	BG2	ST1
2	ST3	L511_w2a	Empty	Empty	L511P_m3	Empty	ST2	ST3	AT809	D516V_w9	Empty	Empty	rpoB9	Empty	Empty	D516V_m1	Empty	Empty	H526_w5	BG1
3	ST1	BG1	H526D_m2	BG2	BG3	H526Y_m5	Empty	Empty	Empty	Empty	S531L_w1	Empty	Empty	S531L_m2	Empty	AT809	ST3	katG6	ST2	ST1
4	ST2	Empty	BG1	Empty	ST1	Empty	BG2	Empty	ST3	Empty	BG3	Empty	ST1	Empty	BG1	Empty	ST2	Empty	BG2	Empty
5	ST1	ST2	S315_w2	AT809	Empty	S315T1_m2	Empty	S315T2_m1	Empty	BG2	BG3	InhA1	BG1	Empty	InhA_w3	Empty	BG3	InhA-15C>T_m3	BG2	ST1
6	ST3	InhA-8T>A_m2	Empty	Empty	InhA-8T>C_m2	Empty	ST2	ST3	AT809	MYC4a	MYC5a	Empty	Empty	MYC6a	Empty	MYC8a	Empty	Empty	MYC10a	BG1
7	ST1	BG1	MYC17a	BG2	BG3	Empty	MYC19a	Empty	MYC16a	Empty	Empty	MYC31a	Empty	Empty	MYC15a	AT809	ST3	Empty	ST2	ST1
8	ST1	ST2	H526_w14	AT809	Empty	rpoB8	Empty	Empty	rpoB14	BG2	BG3	Empty	BG1	Empty	Empty	Empty	BG3	Empty	BG2	ST1
9	ST3	L511_w2a	Empty	Empty	L511P_m3	Empty	ST2	ST3	AT809	D516V_w9	Empty	Empty	rpoB9	Empty	Empty	D516V_m1	Empty	Empty	H526_w5	BG1
10	ST1	BG1	H526D_m2	BG2	BG3	H526Y_m5	Empty	Empty	Empty	Empty	S531L_w1	Empty	Empty	S531L_m2	Empty	AT809	ST3	katG6	ST2	ST1

	21	22	23	24	25	26	27	28	29	30	31	32	33	34	35	36	37	38	39	40
1	ST1	ST2	S315_w2	AT809	Empty	S315T1_m2	Empty	S315T2_m1	Empty	BG2	BG3	InhA1	BG1	Empty	InhA_w3	Empty	BG3	InhA-15C>T_m3	BG2	ST1
2	ST3	InhA-8T>A_m2	Empty	Empty	InhA-8T>C_m2	Empty	ST2	ST3	AT809	MYC4a	MYC5a	Empty	Empty	MYC6a	Empty	MYC8a	Empty	Empty	MYC10a	BG1
3	ST1	BG1	MYC17a	BG2	BG3	Empty	MYC19a	Empty	MYC16a	Empty	Empty	MYC31a	Empty	Empty	MYC15a	AT809	ST3	Empty	ST2	ST1
4	Empty	ST2	Empty	BG1	Empty	ST1	Empty	BG2	Empty	ST3	Empty	BG3	Empty	ST1	Empty	BG1	Empty	ST2	Empty	BG2
5	ST1	ST2	H526_w14	AT809	Empty	rpoB8	Empty	Empty	rpoB14	BG2	BG3	Empty	BG1	Empty	Empty	Empty	BG3	Empty	BG2	ST1
6	ST3	L511_w2a	Empty	Empty	L511P_m3	Empty	ST2	ST3	AT809	D516V_w9	Empty	Empty	rpoB9	Empty	Empty	D516V_m1	Empty	Empty	H526_w5	BG1
7	ST1	BG1	H526D_m2	BG2	BG3	H526Y_m5	Empty	Empty	Empty	Empty	S531L_w1	Empty	Empty	S531L_m2	Empty	AT809	ST3	katG6	ST2	ST1
8	ST1	ST2	S315_w2	AT809	Empty	S315T1_m2	Empty	S315T2_m1	Empty	BG2	BG3	InhA1	BG1	Empty	InhA_w3	Empty	BG3	InhA-15C>T_m3	BG2	ST1
9	ST3	InhA-8T>A_m2	Empty	Empty	InhA-8T>C_m2	Empty	ST2	ST3	AT809	MYC4a	MYC5a	Empty	Empty	MYC6a	Empty	MYC8a	Empty	Empty	MYC10a	BG1
10	ST1	BG1	MYC17a	BG2	BG3	Empty	MYC19a	Empty	MYC16a	Empty	Empty	MYC31a	Empty	Empty	MYC15a	AT809	ST3	Empty	ST2	ST1

Figure S2 HDMA-BG layout 400 spots (replicates: ST1 28 spots, ST2 22 spots, ST3 20 spots, BG1 22 spots, BG2 22 spots BG3 20 spots empty 20, 3 replicas for each MTB specific probe

SI2 Reagents and methods

Chemicals. Glycidoxypropriltrimethoxysilane (GOPS), Sodium Dodecyl Sulfate (SDS), 3 Sodium chloride $mol \cdot L^{-1}$, Sodium Citrate 0.3 $mol \cdot L^{-1}$, Na_2HPO4, KCl, HCl 37%M, CH3COONa, CH_3COONH_4, tris[hydroxymethyl]aminomethane and NaOH, have been purchased from Sigma Adrich. Bovine serum albumin (BSA) has been purchased from Euroclone (EMR086500).

The PCR chemicals (dNTPs, buffer and Hotstart Taq plus DNA polymerase) were purchased from Qiagen (Hilden, Germany). The forward (primer-F) and reverse (primer-R) primers were purchased from MWG (Ebersberg, Germany). Sterile water was purchased from Invitrogen Corp. (Carlsband, CA) and human beta-globin (HBG) was purchased from Jena Bioscience (Jena, Germany). The PCR labelling was performed using dCTP-Cy5 as reported below. To monitor the DNA amplification, the HBB gene was used as DNA template. The target gene sequence is available at the "National Centre for Biotechnology Information" (NCBI) database.

PCR-primers design and selection

The complete HBG gene was 1920 bp in length and consist of 3 small exons and 2 introns. In order to prevent the formation of stable secondary structures like hairpin, self dimer or cross dimer, the primers were designed with ΔG values higher than −2 for hairpin, and higher than −5 for self dimer and cross dimer. The upstream forward primer was designed as complementary to the antisense strand of the β-globin gene (position 72 through 90) while the downstream reverse primer complemented the sense strand (from position 294 to 277). The sequences of PCR primers for BG-gene were: Reverse-primer: 5'- CAGGGCAGTAACGGCAGA-3' (18 bases); Forward-primer: 5'-CTTCTCGGTTCCTGTCCAT-3' (19 bases)

Microarray hybridization reagent

In this specific application for the detection of the HBG gene, the printed microarray layout is composed of hybridization control (ST1, ST2 and ST3) and the specific probe (BG1, BG2 and BG3). The hybridization mix is a mixture of the 250 nM spike-in fluorescently label hybridization control and the 2×hybridization buffer

Table S1. Probe and target oligonucleotides sequences

Oligonucleotides	Probes sequence	Target sequence
BG1	C_6-amino-5'- GCAGAGCCATCTATTGCTTAC-3'	Cy5-5'-GTAAGCAATAGATGGCTCTGC-3'
BG3	C_6-amino- 5'- CTAGGGTTGGCCAATCTACTC-3'	Cy5-5'-GAGTAGATTGGCCAACCCTAG
BG2	C_6-amino- CATCACTTAGACCTCACCCTG-3'	Cy5-5'-CAGGGTGAGGTCTAAGTGATG-3'
ST1	C_6-amino- AGTGAGGGAGGAGATGGAACCATCT-3'	Cy5-5'-AGATGGTTCCATCTCCTCCCTCACT-3'
ST2	C6-amino-5′-CACAACACAAGTACCTGACATGGCG-3′	Cy5-5′-CGCCATGTCAGGTACTTGTGTTGTG-3′
ST3	C6-amino-5′-TGGTCTTCTTAAAAGATTAGTAGGT-3′	Cy5-5′-ACCTACTAATCTTTTAAGAAGACCA-3′

SI3 Fully integrated procedure

In order to perform the fully integrated process (PCR amplification and microarray hybridization), 11.5 —— l of sample was loaded on LoC silicon microreactors by pipettes. Both inlets and outlets were sealed with the PDMS-clamps. The LoCs were located on the thermos-cycler-system (TCS) module before starting thermal sample preparation protocol. Next, the PCR products were moved from the silicon microreactors to the microarray chamber, pipetting 11.5 l of hybridization mix using a gilson P20 pipette and the "Axygen". The LoC was then sealed by specific PDMS-clamps, while the chip was inserted into the TCS and thermally cycled. The PCR/hybridization master mix was composed of 7.5 U Hotstart Taq plus DNA polymerase, 50M dATP, dGTP and dTTP, 25M dCTP, 0.25 M primer-F, 1.0 M primer-R, 1× PCR buffer, 25 M dCTP-Cy5, 70 nM hybridization control AT and sterile water. PCR and hybridization conditions were previously optimized in terms of thermal protocol and reagent concentrations.

The thermal protocol for the BG integrated process consists of: 1) initial denaturation step at 95 ◦C for 5 min, 2) 35 cycles of amplification (20 s at 95 ◦C, 45 s at 61 ◦C, 72 s at 30 ◦C), 3) denaturation step at 95 ◦C for 2 min and 4) hybridization step at 55 ◦C for 30 min. After the thermal process the LoC was removed from the TCS, and a washing step was performed in a centrifuge station, for 5 min at 40 C in 2×SSC. The chip was dried under nitrogen and the image was acquired by the In-Check OR at different exposure time setting.

SI4 Instrumentation

Contact Angle. Aqueous contact angles were measured using a Cam 200 KSV instrument under ambient conditions by using water drops of 1ul. The rate of drop dispensing was 0.5 uL/s. The measures were performed on a control slide (*vide supra*) processed in the same batch of LoC. Three drops were dispensed onto such a slide along a diagonal. For each drop, two contact angle values were measured (left and right) for a total of six values for slide. Environment: 1000 Class. The control slide (2.5 x 7 cm) consists in a silicon substrate covered by layers of Al (1um) and TEOS (850 nm), respectively.

Ellipsometer. The instrument used was a variable angle ellipsometry system SE850 from Sentech. For the measurements the optical constant of SiO_2 layer was set to 1.430 (25°C). This value was previously measured by using a known SiO_2 layer..

XPS. Spectra from X ray photoelectron Spectroscopy (XPS) were acquired under vacuum ($2x10^{-9}$ Torr) with a PHI ESCA/SAM 5600 Multitechnique equipped with a monochromatic source of Al Kpass energy of 11.75 eV and an acceptance angle of ± 7° and several take-off angles ($\theta_{take-off}$ = 10°, 80°)

SI5 Illumination uniformity test.

Figure S3 reports the intensity level profile for the while microarray detection area of LoC-MA (5 mm x 1.25 mm).

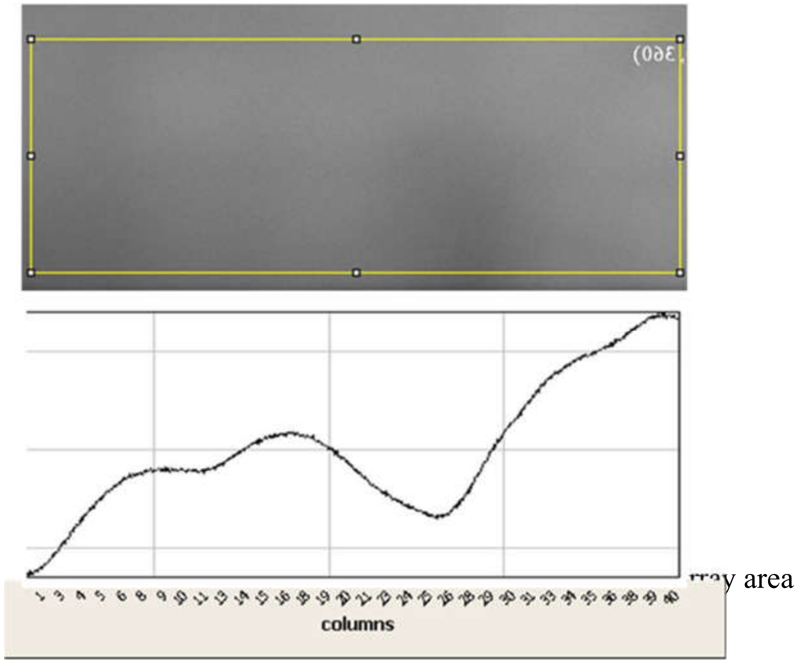

[illegible]rray area

SI6 Calibration curve for spot-probe density.

The calibration curve was obtained using the seven standard solutions here reported. A volume of about 300 pl of each standard solutions have been printed on silanised LoC microarray area.

1. 500 nm of Cy5-labeled oligo probes in Na_2HPO_4 buffer 150 mM (pH=9) adjusted to final concentration of DNA-oligonucleotides of 10 μM with not labeled oligo probe
2. 200 nm of Cy5-labeled oligo probes in Na_2HPO_4 buffer 150 mM (pH=9) adjusted to final concentration of DNA-oligonucleotides of 10 μM with not labeled oligo probe
3. 100 nm of Cy5-labeled oligo probes in Na_2HPO_4 buffer 150 mM (pH=9) adjusted to final concentration of DNA-oligonucleotides of 10 μM with not labeled oligo probe
4. 80 nm of Cy5-labeled oligo probes in Na_2HPO_4 buffer 150 mM (pH=9) adjusted to final concentration of DNA-oligonucleotides of 10 μM with not labeled oligo probe
5. 50 nm of Cy5-labeled oligo probes in Na_2HPO_4 buffer 150 mM (pH=9) adjusted to final concentration of DNA-oligonucleotides of 10 μM with not labeled oligo probe
6. 30 nm of Cy5-labeled oligo probes in Na_2HPO_4 buffer 150 mM (pH=9) adjusted to final concentration of DNA-oligonucleotides of 10 μM with not labeled oligo probe
7. 10 nm of Cy5-labeled oligo probes in Na_2HPO_4 buffer 150 mM (pH=9) adjusted to final concentration of DNA-oligonucleotides of 10 μM with not labeled oligo probe

SI7 Spot size from fluorescence integrated signals for the layout BG-MTB

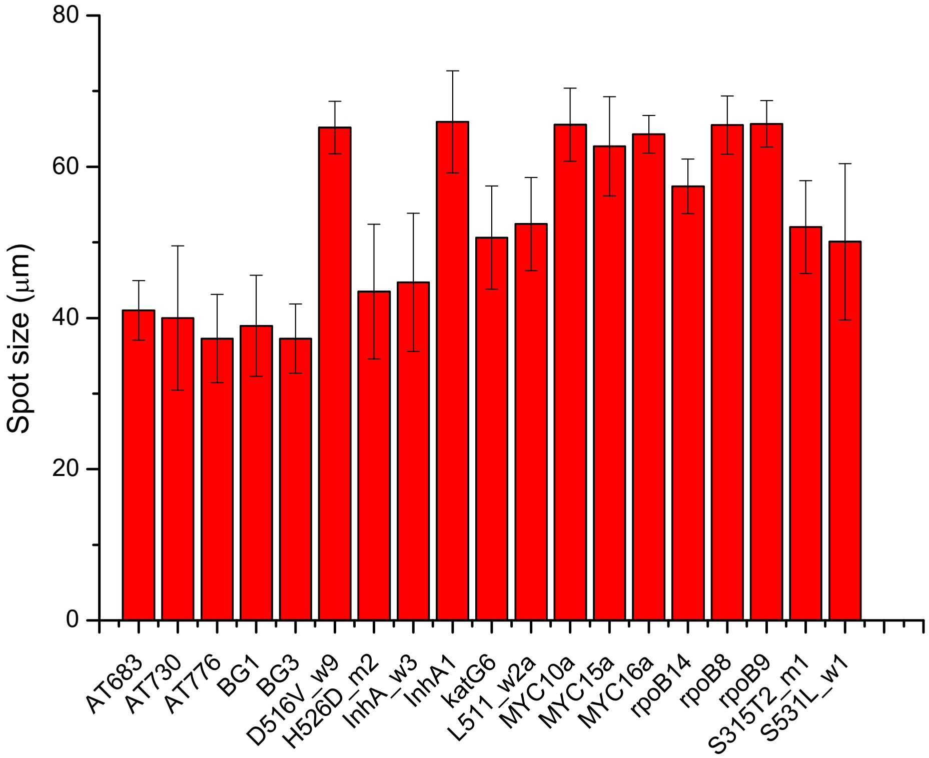

Figure S4 Spot size after Integrated (PCR/hybridization) test

SI8 LoC-MA testing process flow

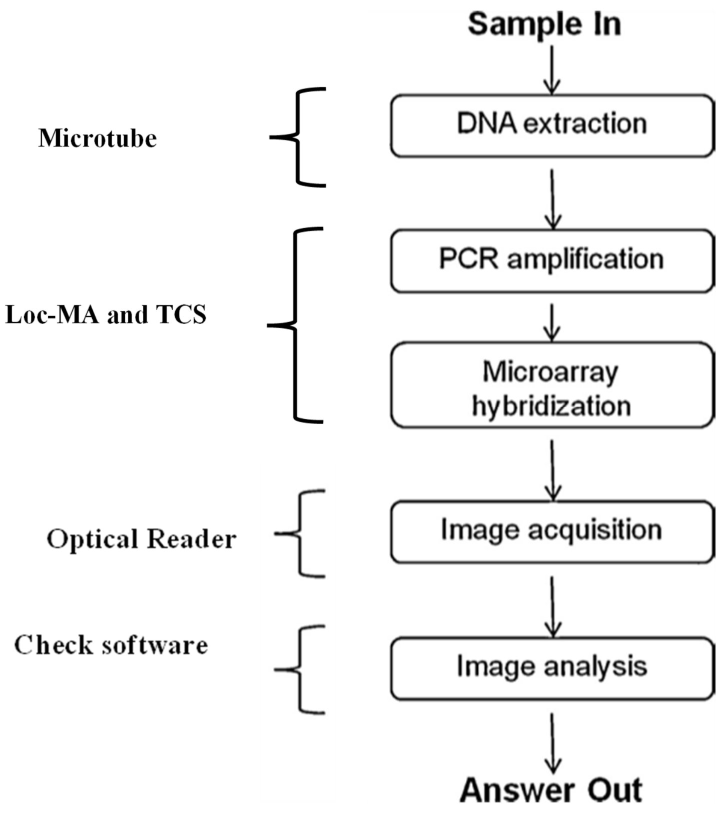

Figure S5 Testing process flow: DNA extraction on tube, PCR amplification and hybridization on LoC-MA, image acquisition by Optical Reader and image analysis.
